# Supplementary material for: Elementary vectors and autocatalytic sets for resource allocation in next-generation models of cellular growth
Source: PLoS Comput Biol. 2022 Feb 1;18(2):e1009843. doi: 10.1371/journal.pcbi.1009843 (PMC8853647; doi:10.1371/journal.pcbi.1009843)
Supplement: S1 Text — Supporting information in four sections: A. The dynamic model of cellular growth. We derive the dynamic model of cellular growth. B. Example: membrane constraints. For the running example (the small model of a self-fabricating cell), we derive the membrane constraints. C. Example: figures and tables. For the running example, we provide supplementary figures and tables. D. Minimal growth model with alternative pathways. We consider a minimal growth model with alternative pathways and provide a complete mathematical analysis of both a constraint-based and a (semi-)kinetic model. (PDF) [file pcbi.1009843.s001.pdf]

Elementary vectors and autocatalytic sets for resource allocation in next-generation models of cellular growth

Stefan Müller[1*], Diana Széliová[2], Jürgen Zanghellini[2,3*]

1 Faculty of Mathematics, University of Vienna, Austria **2** Department of Analytical Chemistry, University of Vienna, Austria **3** Austrian Centre of Industrial Biotechnology, Vienna, Austria

* st.mueller@univie.ac.at (SM), juergen.zanghellini@univie.ac.at (JZ)

Supporting Information

A The dynamic model of cellular growth

We derive the dynamic model of cellular growth (1), studied in the main text.

We denote fundamental objects and quantities as follows:

Mol	set of molecular species	
Rxn	set of chemical reactions	
$N \in \mathbb{R}^{\mathsf{Mol}\times\mathsf{Rxn}}$	stoichiometric matrix	(unit: 1)
$\omega \in \mathbb{R}^{\mathsf{Mol}}_{>}$	molar masses	(unit: $\mathrm{g\,mol^{-1}}$)
$X \in \mathbb{R}^{\mathsf{Mol}}_{\geq}$	amounts of substance	(unit: mol)
$R(\cdot) \in \mathbb{R}^{\mathsf{Rxn}}$	reaction rates (extensive)	(unit: $\mathrm{mol\,h^{-1}}$)

The chemical reactions induce the dynamical system

$$\frac{\mathrm{d}X}{\mathrm{d}t} = NR. \tag{1}$$

We define mass,

$$M = \sum_i \omega_i X_i = \omega^T X, \quad \text{(unit: g)} \tag{2a}$$

the intensive quantities

$$x = \frac{X}{M} \in \mathbb{R}^{\mathsf{Mol}}_{\geq}, \quad (\text{unit: } \mathrm{mol\,g^{-1}}) \tag{2b}$$

$$v = \frac{R}{M} \in \mathbb{R}^{\mathsf{Rxn}}, \quad (\text{unit: } \mathrm{mol\,g^{-1}\,h^{-1}}) \tag{2c}$$

and growth rate

$$\mu = \frac{1}{M}\frac{\mathrm{d}M}{\mathrm{d}t}. \quad (\text{unit: } \mathrm{h^{-1}}) \tag{2d}$$

Thereby, we use mass instead of volume to define the "concentrations" x, the (intensive) reaction rates v, and growth rate μ. In practice, cellular composition is often given in the unit $\mathrm{mol\,g^{-1}}$ (dry weight).

Finally, we recall the chain rule (of differentiation),

$$\frac{\mathrm{d}}{\mathrm{d}t}\frac{X}{M} = \frac{1}{M}\frac{\mathrm{d}X}{\mathrm{d}t} - \frac{X}{M^2}\frac{\mathrm{d}M}{\mathrm{d}t}.$$

Equations (1), (2), and the chain rule yield the dynamic model of cellular growth:

$$\frac{\mathrm{d}x}{\mathrm{d}t} = N v(x) - \mu\, x$$

and

$$\omega^T x = 1.$$

Thereby, we assume given cell density. Recall that reaction rates depend on (volumetric) concentrations X/V,

$$R = R(X/V) = R(M/V \cdot X/M) = R(\rho\, x)$$

with volume V (unit: L) and cell density $\rho = \frac{M}{V}$ (unit: $\mathrm{g\,L^{-1}}$). Hence,

$$v = \frac{R}{M} = \frac{R(\rho\, x)}{M} = v(\rho\, x).$$

For constant cell density ρ, $v = v(x)$ only depends on concentrations.

For alternative derivations, see e.g. [2] or [1].

By multiplying the mass balance equation with a vector $c \in \mathbb{R}^{\mathsf{Mol}}$, we obtain

$$\frac{\mathrm{d}(c^T x)}{\mathrm{d}t} = c^T N v(x) - \mu\,(c^T x).$$

We highlight two observations that hold for any model of cellular growth.

Fact (conservation laws). In a model of cellular growth, there cannot be any conservation laws. In mathematical terms, $\ker N^T \cap \mathbb{R}^{\mathsf{Mol}}_{\geq} = \{0\}$.

To see this, assume $c^T N = 0$ with $0 \neq c \geq 0$, for example, assume $c_1 = c_2 = 1$ and $c_i = 0$, otherwise. Then, $\frac{\mathrm{d}(c^T x)}{\mathrm{d}t} = \frac{\mathrm{d}(x_1 + x_2)}{\mathrm{d}t} = -\mu(x_1 + x_2) \leq 0$, and $\mu > 0$ implies $x_1 = x_2 = 0$ at steady state.

Fact (dependent concentrations). In a model of cellular growth, there can be dependent concentrations. In mathematical terms, $\ker N^T \neq \{0\}$.

To see this, assume $c^T N = 0$ with $0 \neq c$, for example, assume $c_1 = 1$, $c_2 = -1$, and $c_i = 0$, otherwise. Then, $\frac{\mathrm{d}(c^T x)}{\mathrm{d}t} = \frac{\mathrm{d}(x_1 - x_2)}{\mathrm{d}t} = -\mu(x_1 - x_2)$, and $\mu > 0$ implies $x_1 = x_2$ at steady state.

B Example: membrane constraints

For the small model of a self-fabricating cell studied in the main text, we derive the membrane constraints (7c) and (7d).

The cell membrane area A is formed by lipids L and importers IG and IN,

$$A = A_{\mathsf{L}} \cdot \#\mathsf{L} + A_{\mathsf{I}} \cdot (\#\mathsf{IG} + \#\mathsf{IN}),$$

where A_{L} and A_{I} denote the areas of lipids and importers, respectively, and $\#X$ denotes the number of molecule X. After division by Avogadro's number N_A, we have

$$\frac{A}{N_A} = A_{\mathsf{L}}\, s_{\mathsf{L}} + A_{\mathsf{I}}\,(s_{\mathsf{IG}} + s_{\mathsf{IN}}),$$

where $s_X = \frac{\#X}{N_A}$ denotes the amount of substance. Further, after division by cell mass m, we have

$$\frac{A}{m\, N_A} = A_{\mathsf{L}}\, x_{\mathsf{L}} + A_{\mathsf{I}}\,(x_{\mathsf{IG}} + x_{\mathsf{IN}}),$$

where $x_X = \frac{s_X}{m}$ denotes the (mass-specific) concentration. Finally, using cell volume V, the surface-to-volume ratio $r = \frac{A}{V}$, and cell density $\rho = \frac{m}{V}$, we obtain $\frac{A}{m} = \frac{A}{V}\frac{V}{m} = \frac{r}{\rho}$ and hence

$$\frac{r}{\rho\, N_A} = A_{\mathsf{L}}\, x_{\mathsf{L}} + A_{\mathsf{I}}\,(x_{\mathsf{IG}} + x_{\mathsf{IN}}).$$

Additionally, we require that a minimum fraction α of the surface area is formed by lipids,

$$A_{\mathsf{L}} \cdot \#\mathsf{L} \geq \alpha\, A = \alpha\,(A_{\mathsf{L}} \cdot \#\mathsf{L} + A_{\mathsf{I}} \cdot (\#\mathsf{IG} + \#\mathsf{IN}))\,,$$

that is,

$$A_{\mathsf{L}}\, x_{\mathsf{L}} \geq \alpha\,(A_{\mathsf{L}}\, x_{\mathsf{L}} + A_{\mathsf{I}}\,(x_{\mathsf{IG}} + x_{\mathsf{IN}}))\,,$$

where we use concentrations instead of numbers of molecules. Equivalently,

$$(1-\alpha)\, A_{\mathsf{L}}\, x_{\mathsf{L}} \geq \alpha\, A_{\mathsf{I}}\,(x_{\mathsf{IG}} + x_{\mathsf{IN}}).$$

C Example: figures and tables

**Name**	**(In)equality**
mass balance G	$v_{\mathsf{IG}} - v_{\mathsf{EAA}} - n_{\mathsf{L}} v_{\mathsf{ELD}} \geq 0$
mass balance N	$v_{\mathsf{IN}} - v_{\mathsf{EAA}} \geq 0$
mass balance AA	$v_{\mathsf{EAA}} - n_{\mathsf{I}}\,(w_{\mathsf{IG}} + w_{\mathsf{IN}}) - n_{\mathsf{E}}\,(w_{\mathsf{EAA}} + w_{\mathsf{ELD}} + w_{\mathsf{EL}}) - n_{\mathsf{R}}\, w_{\mathsf{R}} \geq 0$
mass balance LD	$v_{\mathsf{ELD}} - v_{\mathsf{EL}} \geq 0$
capacity IG	$v_{\mathsf{IG}} \leq k_{\text{cat}}\, x_{\mathsf{IG}}$
capacity IN	$v_{\mathsf{IN}} \leq k_{\text{cat}}\, x_{\mathsf{IN}}$
capacity EAA	$v_{\mathsf{EAA}} \leq k_{\text{cat}}\, x_{\mathsf{EAA}}$
capacity ELD	$v_{\mathsf{ELD}} \leq k_{\text{cat}}\, x_{\mathsf{ELD}}$
capacity EL	$v_{\mathsf{EL}} \leq k_{\text{cat}}\, x_{\mathsf{EL}}$
capacity R	$n_{\mathsf{I}}\,(w_{\mathsf{IG}} + w_{\mathsf{IN}}) + n_{\mathsf{E}}\,(w_{\mathsf{EAA}} + w_{\mathsf{ELD}} + w_{\mathsf{EL}}) + n_{\mathsf{R}}\, w_{\mathsf{R}} \leq k_{\text{el}}\, x_{\mathsf{R}}$
membrane L	$(1-\alpha)\, A_{\mathsf{L}}\, x_{\mathsf{L}} \geq \alpha\, A_{\mathsf{I}}\,(x_{\mathsf{IG}} + x_{\mathsf{IN}})$
membrane	$A_{\mathsf{L}}\, x_{\mathsf{L}} + A_{\mathsf{I}}\,(x_{\mathsf{IG}} + x_{\mathsf{IN}}) = \frac{r}{\rho\, N_A}$
(dry) mass	$\omega^T x = 1$

Table A. Essential constraints for the small model of a self-fabricating cell.

Mass balance constraints $(Nv)_s \geq 0$ are stated only for metabolites $s \in \{\mathsf{G}, \mathsf{N}, \mathsf{AA}, \mathsf{LD}\}$, since lipids L and macromolecules $\mathsf{Mac} = \{\mathsf{IG}, \mathsf{IN}, \mathsf{EAA}, \mathsf{ELD}, \mathsf{EL}, \mathsf{R}\}$ are products of exactly one reaction each and not educts of any reaction. The corresponding constraints $v_{\mathsf{EL}} \geq 0$ and $w_s \geq 0$ for $s \in \mathsf{Mac}$ are covered by the irreversibility of all reactions, $v \geq 0$. The last two constraints are *equality* constraints (which are always active).

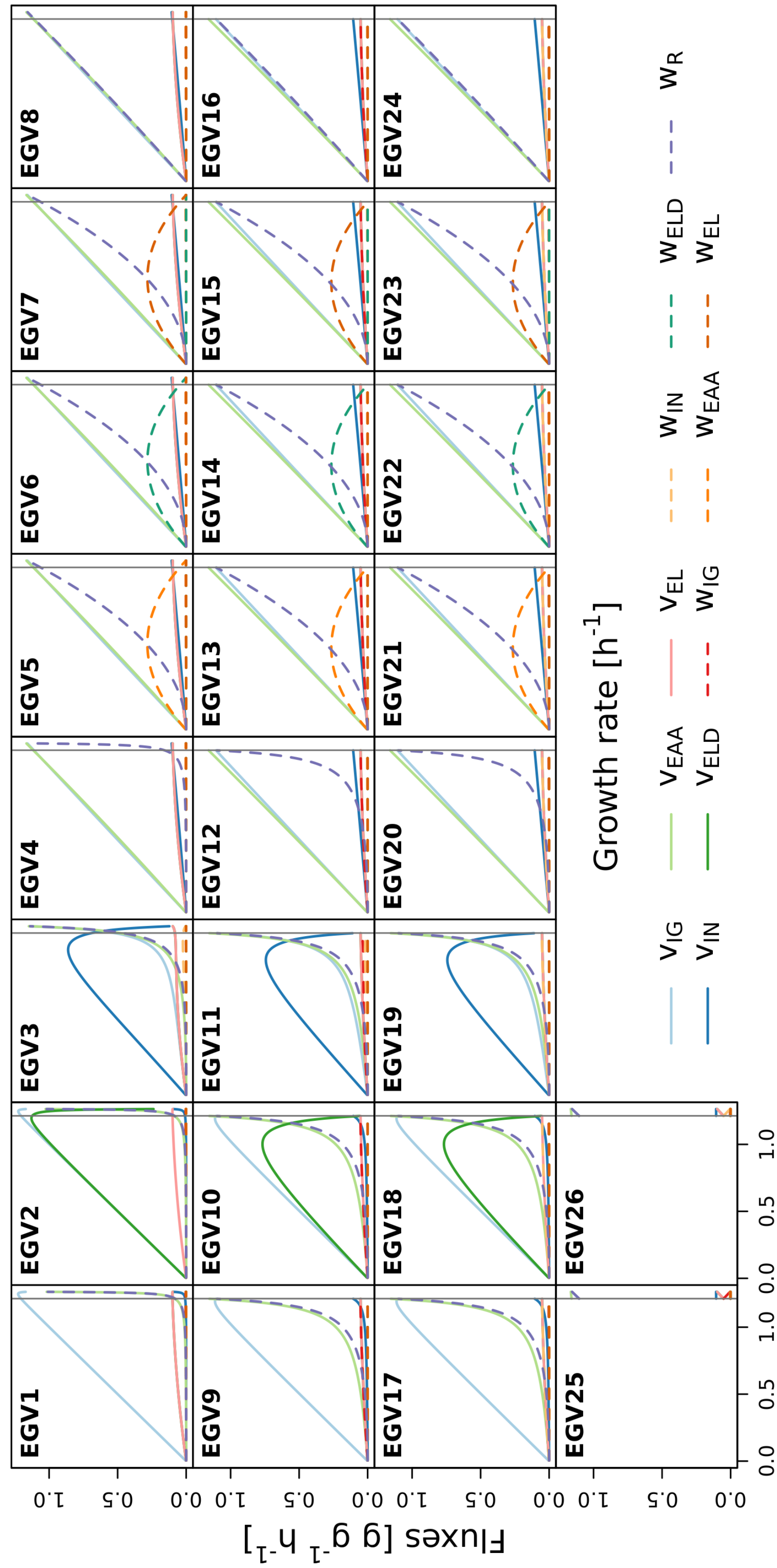

Fig A. (Scaled) fluxes for all 26 EGVs.

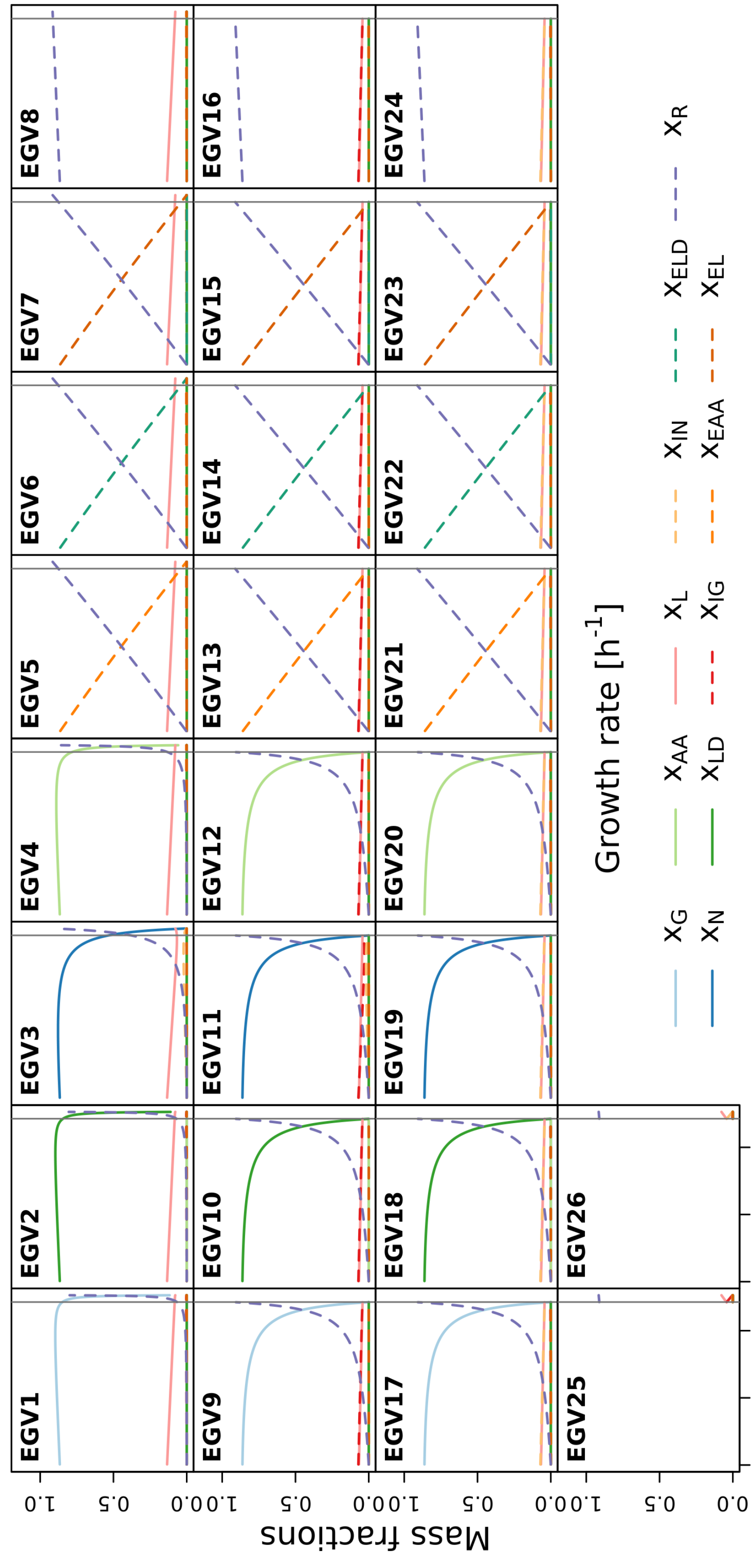

Fig B. Mass fractions for all 26 EGVs.

(a)

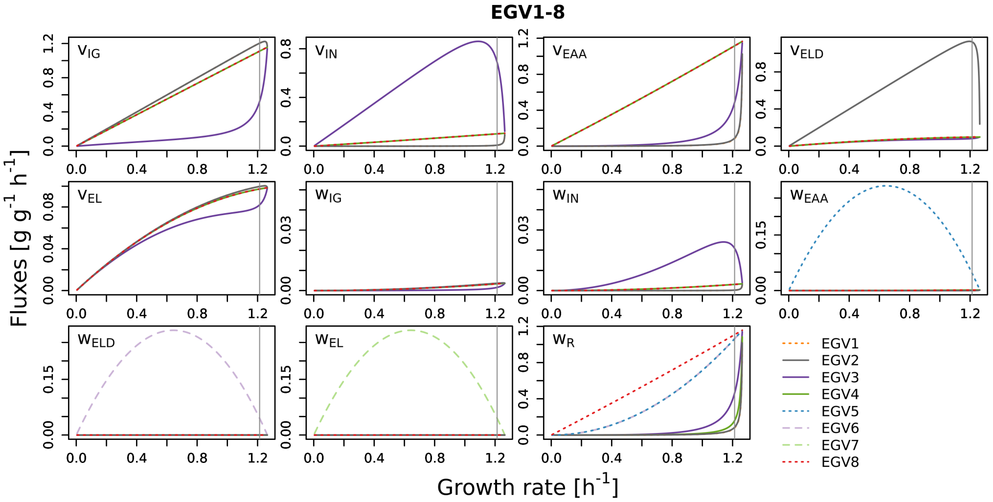

(b)

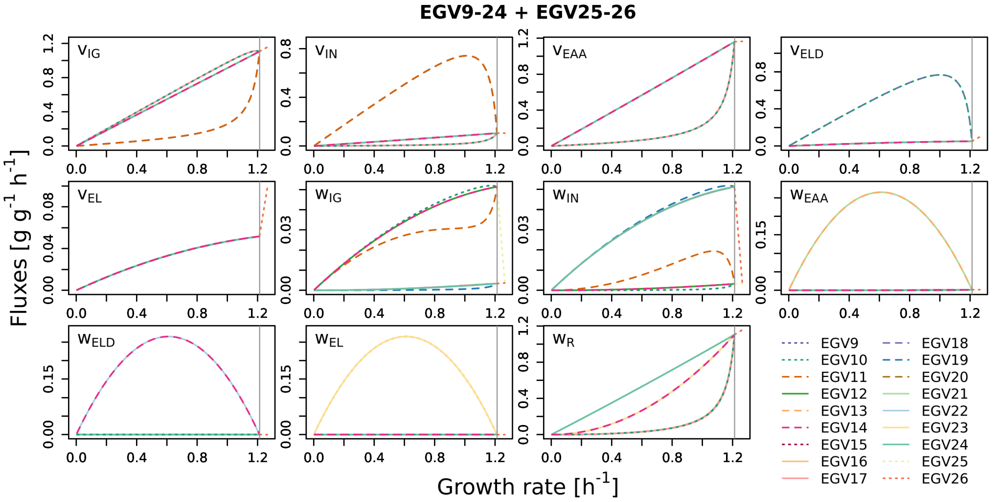

Fig C. (Scaled) fluxes for (a) the 8 EGVs that exist for all growth rates and (b) the 16 EGVs that exist in regime L plus the 2 EGVs that exist in regime H.

D Minimal growth model with alternative pathways

Consider the following minimal model of cellular growth with two alternative pathways:

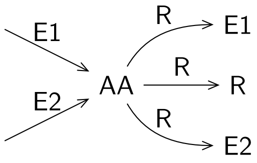

The cell takes up external substrates and forms amino acids (AA) via two "reactions",

$$r_1 : \ \overset{\mathsf{E1}}{\to} \mathsf{AA} \quad \text{and} \quad r_2 : \ \overset{\mathsf{E2}}{\to} \mathsf{AA},$$

catalyzed by the "enzymes" E1 and E2, respectively. Amino acids are then used by the ribosome (R) to synthesize the enzymes and the ribosome itself,

$$s_1 : \ \mathsf{AA} \overset{\mathsf{R}}{\to} \mathsf{E1}, \quad s_2 : \ \mathsf{AA} \overset{\mathsf{R}}{\to} \mathsf{E2}, \quad s_{\mathsf{R}} : \ \mathsf{AA} \overset{\mathsf{R}}{\to} \mathsf{R}.$$

The set of molecular species is $\mathsf{Mol} = \{\mathsf{AA}, \mathsf{E1}, \mathsf{E2}, \mathsf{R}\}$, and the set of reactions is $\mathsf{Rxn} = \mathsf{Rmet} \cup \mathsf{Rsyn}$ with metabolic reactions $\mathsf{Rmet} = \{r_1, r_2\}$ and synthesis reactions $\mathsf{Rsyn} = \{s_1, s_2, s_{\mathsf{R}}\}$.

The resulting stoichiometric matrix and the corresponding flux vector are given by

$$\begin{array}{c} \\ \mathsf{AA} \\ \mathsf{E1} \\ \mathsf{E2} \\ \mathsf{R} \end{array} \begin{array}{c} \begin{array}{ccccc} r_1 & r_2 & s_1 & s_2 & s_{\mathsf{R}} \end{array} \\ \begin{pmatrix} 1 & 1 & -1 & -1 & -1 \\ 0 & 0 & 1 & 0 & 0 \\ 0 & 0 & 0 & 1 & 0 \\ 0 & 0 & 0 & 0 & 1 \end{pmatrix} \end{array} = N$$

and

$$(v_1,\, v_2;\, w_1,\, w_2,\, w_{\mathsf{R}})^T = v.$$

By mass conservation (for the synthesis reactions), the molar masses obey $\omega = \bar{\omega} \cdot (1, 1, 1, 1)^T$. The growth cone is given by

$$C_{\mathrm{g}} = \{(v_1, v_2; w_1, w_2, w_{\mathsf{R}})^T \in \mathbb{R}^{\mathsf{Rxn}}_{\geq} \mid v_1 + v_2 - w_1 - w_2 - w_{\mathsf{R}} \geq 0\}.$$

As it turns out, there are $4 \times 2 = 8$ EGMs (up to scaling), corresponding to the four species and the two alternative pathways, that is, $e^{s,i} \in \mathbb{R}^{\mathsf{Rxn}}_{\geq}$ with $s \in \mathsf{Mol}$ and $i \in \{1, 2\}$. Explicitly,

$$\begin{aligned} e^{\mathsf{AA},1} &= \mu/\bar{\omega} \cdot (1, 0; 0, 0, 0)^T, \\ e^{\mathsf{AA},2} &= \mu/\bar{\omega} \cdot (0, 1; 0, 0, 0)^T, \\ e^{\mathsf{E1},1} &= \mu/\bar{\omega} \cdot (1, 0; 1, 0, 0)^T, \\ e^{\mathsf{E1},2} &= \mu/\bar{\omega} \cdot (0, 1; 1, 0, 0)^T, \\ e^{\mathsf{E2},1} &= \mu/\bar{\omega} \cdot (1, 0; 0, 1, 0)^T, \\ e^{\mathsf{E2},2} &= \mu/\bar{\omega} \cdot (0, 1; 0, 1, 0)^T, \\ e^{\mathsf{R},1} &= \mu/\bar{\omega} \cdot (1, 0; 0, 0, 1)^T, \\ e^{\mathsf{R},2} &= \mu/\bar{\omega} \cdot (0, 1; 0, 0, 1)^T. \end{aligned}$$

Every EGM "produces" exactly one molecular species, as indicated by its name, thereby using either pathway 1 or 2. (For every EGM, there is exactly one species with nonzero associated concentration.) Due to the factor $\mu/\bar{\omega}$, all EGMs have associated growth rate μ.

GMs need not be AC, in particular, no EGM is AC. In fact, every (nonzero) GM is BC, since all reactions are catalytic, however, a GM need not be CC. MAC subsets of reactions are the supports of AC GMs. There are two MAC sets, namely $M_1 = \{r_1, s_1, s_\mathsf{R}\}$ and $M_2 = \{r_2, s_2, s_\mathsf{R}\}$, corresponding to the two alternative pathways. AC GMs with support M_1 are generated by the EGMs $e^{\mathsf{AA},1}, e^{\mathsf{E1},1}, e^{\mathsf{R},1}$. (Analogously for the MAC set M_2.)

For a GM $v \in C_\mathrm{g}$, the associated growth rate amounts to

$$\mu = \omega^T N \, v = \bar{\omega} \, (v_1 + v_2),$$

determined by the "exchange" fluxes v_1 and v_2. For fixed growth rate μ, the growth cone becomes a growth polytope,

$$P_\mathrm{g}(\mu) = \big\{ (v_1, v_2; w_1, w_2, w_\mathsf{R})^T \in \mathbb{R}^{\mathsf{Rxn}}_{\geq} \,|\, w_1 + w_2 + w_\mathsf{R} \leq v_1 + v_2, \\ v_1 + v_2 = \mu/\bar{\omega} \ \big\} \, .$$

Further, for scaled fluxes $\hat{v} = \bar{\omega}/\mu \cdot v$, the polytope becomes independent of μ,

$$\hat{P}_\mathrm{g} = \big\{ (\hat{v}_1, \hat{v}_2; \hat{w}_1, \hat{w}_2, \hat{w}_\mathsf{R})^T \in \mathbb{R}^{\mathsf{Rxn}}_{\geq} \,|\, \hat{w}_1 + \hat{w}_2 + \hat{w}_\mathsf{R} \leq 1, \\ \hat{v}_1 + \hat{v}_2 = 1 \ \big\} \, .$$

In particular, its projection to the synthesis fluxes $\hat{w} \in \mathbb{R}^{\mathsf{Rsyn}}_{\geq}$ is the "growth simplex"

$$\mathcal{P}_\mathrm{g} = \{ (\hat{w}_1, \hat{w}_2, \hat{w}_\mathsf{R})^T \in \mathbb{R}^{\mathsf{Rsyn}}_{\geq} \mid \hat{w}_1 + \hat{w}_2 + \hat{w}_\mathsf{R} \leq 1 \},$$

spanned by the projections of the scaled EGMs,

$$\begin{aligned} e^{\mathsf{AA}} &= (0,0,0)^T, \\ e^{\mathsf{E1}} &= (1,0,0)^T, \\ e^{\mathsf{E2}} &= (0,1,0)^T, \\ e^{\mathsf{R}} &= (0,0,1)^T. \end{aligned}$$

The "projections" of the MAC sets M_1 and M_2 to the synthesis reactions are $m_1 = \{s_1, s_\mathsf{R}\}$ and $m_2 = \{s_2, s_\mathsf{R}\}$. Scaled projections of AC GMs with support m_1 lie in the (two-dimensional) simplex generated by $e^{\mathsf{AA}}, e^{\mathsf{E1}}, e^{\mathsf{R}}$. (Analogously for m_2.)

Catalytic closure can be ensured by additional constraints.

- In a constraint-based model, one considers *inequality* enzyme capacity constraints,

  $$v_i \leq k_i^{\mathrm{cat}} \, x_{\mathsf{E}i}$$

  for $i \in \{1,2\}$, whereas

- in a (semi-)kinetic model, one considers *equality* constraints arising from enzyme and ribosome kinetics,

  $$v_i = \kappa_i(x_\mathsf{AA}) \, x_{\mathsf{E}i} \quad \text{and} \quad w_j = \alpha_j \, \tau_j(x_\mathsf{AA}) \, x_\mathsf{R}$$

  for $i \in \{1,2\}$ and $j \in \{1,2,\mathsf{R}\}$. Thereby, κ_i, τ_j are functions of the amino acid concentration x_AA, and α_j are control parameters (ribosome fractions) for studying growth rate maximization, cf. [1,3].

Moreover, one often considers ribosome capacity constraints: $w_1 + w_2 + w_{\mathsf{R}} \leq k_{\mathrm{tl}}\, x_{\mathsf{R}}$ in constraint-based models and $\sum_{j\in\{1,2,\mathsf{R}\}} \alpha_j \leq 1$ in (semi-)kinetic models. However, the (inequality) ribosome capacity constraint is treated separately in the (semi-)kinetic model, and for reasons of comparison, we just require $x_{\mathsf{R}} > 0$ in the constraint-based model.

Additional constraints involve concentrations. For a GM $v \in C_{\mathrm{g}}$, the associated concentrations x are given by

$$Nv = (*, w_1, w_2, w_{\mathsf{R}})^T = \mu\, x = \mu\, (x_{\mathsf{AA}}, x_{\mathsf{E1}}, x_{\mathsf{E2}}, x_{\mathsf{R}})^T.$$

In particular, $x_{\mathsf{E1}} = w_1/\mu$ and $x_{\mathsf{E2}} = w_2/\mu$.

In the following, we consider the growth polyhedra and EGVs arising from the additional constraints.

Constraint-based model. For fixed growth rate $\mu > 0$, the growth polytope $P_{\mathrm{g}}(\mu)$ above is further restricted by *inequality* constraints,

$$\begin{aligned} P_{\mathrm{g},\geq}(\mu) = \big\{(v_1, v_2; w_1, w_2, w_{\mathsf{R}})^T \in \mathbb{R}^{\mathsf{Rxn}}_{\geq} \,|\, & w_1 + w_2 + w_{\mathsf{R}} \leq v_1 + v_2, \\ & v_1 + v_2 = \mu/\bar{\omega}, \\ & v_1 \leq k_1^{\mathsf{cat}}\, w_1/\mu,\; v_2 \leq k_2^{\mathsf{cat}}\, w_2/\mu \;\big\}. \end{aligned}$$

For scaled fluxes $\hat{v} = \bar{\omega}/\mu \cdot v$, the polytope becomes

$$\begin{aligned} \hat{P}_{\mathrm{g},\geq}(\mu) = \big\{(\hat{v}_1, \hat{v}_2, \hat{w}_1, \hat{w}_2, \hat{w}_{\mathsf{R}})^T \in \mathbb{R}^{\mathsf{Rxn}}_{\geq} \,|\, & \hat{w}_1 + \hat{w}_2 + \hat{w}_{\mathsf{R}} \leq 1, \\ & \hat{v}_1 + \hat{v}_2 = 1, \\ & \mu\, \hat{v}_1 \leq k_1^{\mathsf{cat}}\, \hat{w}_1,\; \mu\, \hat{v}_2 \leq k_2^{\mathsf{cat}}\, \hat{w}_2 \;\big\}. \end{aligned}$$

Its projection to the synthesis fluxes $\hat{w} \in \mathbb{R}^{\mathsf{Rsyn}}_{\geq}$ is given by

$$\begin{aligned} \mathcal{P}_{\mathrm{g},\geq}(\mu) = \Big\{(\hat{w}_1, \hat{w}_2, \hat{w}_{\mathsf{R}})^T \in \mathbb{R}^{\mathsf{Rsyn}}_{\geq} \,|\, & \hat{w}_1 + \hat{w}_2 + \hat{w}_{\mathsf{R}} \leq 1, \\ & \mu \leq k_1^{\mathsf{cat}}\, \hat{w}_1 + k_2^{\mathsf{cat}}\, \hat{w}_2 \;\big\}. \end{aligned}$$

(Scaled) EGVs are the ccND vectors of a (scaled) growth polytope. (Here, since $\hat{P}_{\mathrm{g},\geq}(\mu)$ is contained in the nonnegative orthant, EGVs are the vertices.) The number of EGVs depends on μ. For small μ, there are 8 EGVs. As it turns out, there are at most two EGVs with nonzero ribosome concentration/flux, namely

$$\begin{aligned} &(1,\ 0;\ \mu/k_1^{\mathsf{cat}},\ 0,\ 1 - \mu/k_1^{\mathsf{cat}})^T, \\ &(0,\ 1;\ 0,\ \mu/k_2^{\mathsf{cat}},\ 1 - \mu/k_2^{\mathsf{cat}})^T. \end{aligned}$$

Only these EGVs can fulfill the additional ribosome capacity constraint. They are AC, and their supports are the MAC sets M_1 and M_2. Their projections are

$$\begin{aligned} e^1 &= (\mu/k_1^{\mathsf{cat}},\ 0,\ 1 - \mu/k_1^{\mathsf{cat}})^T, \\ e^2 &= (0,\ \mu/k_2^{\mathsf{cat}},\ 1 - \mu/k_2^{\mathsf{cat}})^T, \end{aligned}$$

which have supports m_1 and m_2, respectively.

(Semi-)kinetic model. For fixed growth rate $\mu > 0$ and fixed amino acid concentration $x_{\mathsf{AA}} =: \bar{x}$ (and hence fixed functions $\kappa_i(\bar{x}) =: \bar{\kappa}_i$), the growth polytope $P_{\mathrm{g}}(\mu)$ is further restricted by *equality* constraints,

$$P_{\mathrm{g},=}(\mu, \bar{x}) = \big\{ (v_1, v_2; w_1, w_2, w_{\mathsf{R}})^T \in \mathbb{R}^{\mathsf{Rxn}}_{\geq} \,|\, v_1 + v_2 - w_1 - w_2 - w_{\mathsf{R}} = \mu\, \bar{x}, \\ v_1 + v_2 = \mu/\bar{\omega}, \\ v_1 = \bar{\kappa}_1\, w_1/\mu,\ v_2 = \bar{\kappa}_2\, w_2/\mu \,\big\} \, .$$

For scaled fluxes $\hat{v} = \bar{\omega}/\mu \cdot v$, the polytope becomes

$$\hat{P}_{\mathrm{g},=}(\mu, \bar{x}) = \big\{ (\hat{v}_1, \hat{v}_2, \hat{w}_1, \hat{w}_2, \hat{w}_{\mathsf{R}})^T \in \mathbb{R}^{\mathsf{Rxn}}_{\geq} \,|\, \hat{v}_1 + \hat{v}_2 - \hat{w}_1 - \hat{w}_2 - \hat{w}_{\mathsf{R}} = \bar{\omega}\, \bar{x}, \\ \hat{v}_1 + \hat{v}_2 = 1, \\ \hat{v}_1 = \bar{\kappa}_1\, \hat{w}_1/\mu,\ \hat{v}_2 = \bar{\kappa}_2\, \hat{w}_2/\mu \,\big\} \, .$$

Since $\hat{v}_1, \hat{v}_2$ depend on $\hat{w}_1, \hat{w}_2$, the polytope is in one-to-one correspondence with its projection to the synthesis fluxes $\hat{w} \in \mathbb{R}^{\mathsf{Rsyn}}_{\geq}$,

$$\mathcal{P}_{\mathrm{g},=}(\mu, \bar{x}) = \Big\{ (\hat{w}_1, \hat{w}_2, \hat{w}_{\mathsf{R}})^T \in \mathbb{R}^{\mathsf{Rsyn}}_{\geq} \,|\, 1 - \hat{w}_1 - \hat{w}_2 - \hat{w}_{\mathsf{R}} = \bar{\omega}\, \bar{x}, \\ \bar{\kappa}_1\, \hat{w}_1 + \bar{\kappa}_2\, \hat{w}_2 = \mu \,\big\} \, .$$

Again, scaled EGVs are vertices of the scaled polytope $\hat{P}_{\mathrm{g},=}(\mu, \bar{x})$. As it turns out, there are at most two EGVs, in particular, at most two EGVs with nonzero ribosome concentration/flux, namely

$$(1,\ 0;\ \mu/\bar{\kappa}_1,\ 0,\ 1 - \bar{\omega}\, \bar{x} - \mu/\bar{\kappa}_1)^T \, , \\ (0,\ 1;\ 0,\ \mu/\bar{\kappa}_2,\ 1 - \bar{\omega}\, \bar{x} - \mu/\bar{\kappa}_2)^T \, .$$

These EGVs are AC, and their supports are the MAC sets M_1 and M_2. They are in one-to-one correspondence with their projections,

$$\varepsilon^1 = (\mu/\bar{\kappa}_1,\ 0,\ 1 - \bar{\omega}\, \bar{x} - \mu/\bar{\kappa}_1)^T \, , \\ \varepsilon^2 = (0,\ \mu/\bar{\kappa}_2,\ 1 - \bar{\omega}\, \bar{x} - \mu/\bar{\kappa}_2)^T \, ,$$

the vertices of the polytope $\mathcal{P}_{\mathrm{g},=}(\mu, \bar{x})$, which are called *elementary growth states* (EGSs) in [1]. (In fact, EGSs are defined via the control parameters α, which are, however, in one-to-one correspondence with the synthesis fluxes w.)

The supports of the EGSs ε_1 and ε_2, that is, m_1 and m_2, the projections of the MAC sets, are called *elementary growth modes* in [1].

References

1. de Groot DH, Hulshof J, Teusink B, Bruggeman FJ, Planqué R. Elementary Growth Modes provide a molecular description of cellular self-fabrication. PLoS Comput Biol. 2020;16(1):e1007559.

2. de Jong H, Casagranda S, Giordano N, Cinquemani E, Ropers D, Geiselmann J, et al. Mathematical modelling of microbes: metabolism, gene expression and growth. Journal of The Royal Society Interface. 2017;14(136):20170502.

3. Molenaar D, van Berlo R, de Ridder D, Teusink B. Shifts in growth strategies reflect tradeoffs in cellular economics. Mol Syst Biol. 2009;5(1):323.
